# MyD88 Inhibition Ameliorates Diabetes-Induced Hepatic Inflammation and Gluconeogenesis Through Adipose IL-10 Induction

**DOI:** 10.3390/ijms27062883

**Published:** 2026-03-23

**Authors:** Yi-Cheng Li, Hsiao-Chi Lai, Pei-Hsuan Chen, Chia-Hua Tang, Lee-Wei Chen

**Affiliations:** 1 Department of Surgery, Zuoying Armed Forces General Hospital, Kaohsiung 81342, Taiwan; doc82030@gmail.com; 2 Department of Surgery, Kaohsiung Veterans General Hospital, No.386, Ta-Chung 1st Road, Kaohsiung 81362, Taiwan; hclaihd91@vghks.gov.tw (H.-C.L.); hsuan9041@gmail.com (P.-H.C.); chtang@vghks.gov.tw (C.-H.T.); 3 Department of Pharmacy and Master Program, College of Pharmacy and Health Care, Tajen University, No.20, Weixin Road, Pingtung 90741, Taiwan; 4 Institute of Emergency and Critical Care Medicine, National Yang Ming Chiao Tung University, No.155, Sec.2, Linong Street, Taipei 11221, Taiwan; 5 Department of Biological Sciences, National Sun Yat-Sen University, No.70, Lien-Hai Road, Kaohsiung 80424, Taiwan

**Keywords:** adiponectin, stromal vascular fraction, regulatory T cells, DPP4, IL-6, Foxp3

## Abstract

Myeloid differentiation factor 88 (MyD88) signaling plays a central role in inflammatory pathway activation. Adipose-derived interleukin-10 (IL-10), which is induced by insulin and lipopolysaccharides, suppresses hepatic glucose production. This study investigated the role of MyD88/IL-10 signaling in diabetes-induced systemic inflammation and hepatic gluconeogenesis. Stromal vascular fractions (SVFs) were isolated from the adipose tissue of *Lepr^db^*^/*db*^ and *Lepr^db^*^/*db*^MyD88^−/−^ mice and treated with IL-10 followed by analysis of inflammatory cytokine expression. IL-10 (10 or 50 ng) was injected into adipose tissue of type 2 DM (T2DM) (*Lepr^db^*^/*db*^) mice to investigate its effect on blood dipeptidyl peptidase-4 (DPP4) activity, insulin resistance, and hepatic gluconeogenic signaling. Hepatic inflammatory markers, gluconeogenic gene expression, and metabolic parameters were assessed. Compared with wild-type mice, *Lepr^db^*^/*db*^ mice exhibited significantly reduced FOXP3 protein expression and IL-10 levels in adipose tissue, accompanied by increased blood DPP4 activity and adiponectin levels, elevated hepatic inflammatory cytokines, and increased *G6pc* and *Pck1* mRNA expression. In contrast, *Lepr^db^*^/*db*^MyD88^−/−^ mice showed increased Foxp3 protein and *PDGFα* mRNA expression, decreased *IL-6* and *CCL2* mRNA expression in SVFs, increased IL-10 levels in adipose tissue, and lower blood adiponectin and ALT levels. MyD88 deletion also attenuated Kupffer cell accumulation, hepatic inflammatory cytokine expression, and gluconeogenic gene expression. In vitro, IL-10 treatment of SVFs from *Lepr^db^*^/*db*^ mice significantly reduced *IL-6* and *CCL2* expression and increased *Foxp3* mRNA expression. In vivo, adipose IL-10 injection increased *Foxp3* and *IL-10* expression, expanded Treg cells in SVFs, and activated hepatic Akt signaling, while suppressing pJNK and pNF-κB signaling. These changes were accompanied by reduced blood DPP4 activity, ALT and adiponectin levels, decreased Kupffer cell-derived inflammatory cytokines, reduced hepatic *G6pc* and *Pck1* expression, and improved glucose tolerance. MyD88 signaling induces adipose *IL-6* and *CCL2*, liver inflammation and gluconeogenesis, and blood DPP4 activity by reducing IL-10 and Foxp3 of adipose tissue in T2DM. Enhancing adipose IL-10 induces Treg expansion, inhibits JNK and NF-κB signaling, and alleviates hepatic gluconeogenesis and insulin resistance. MyD88 inhibition or IL-10 elevation in adipose tissue may represent a novel strategy for metabolic syndrome.

## 1. Introduction

Obesity and its associated insulin resistance represent major global health challenges and are key contributors to chronic diseases such as cardiovascular disorders and type 2 diabetes mellitus (T2DM). Impaired tissue responsiveness to insulin in obesity has been closely linked to defective insulin signaling driven by systemic low-grade inflammation [1]. Accumulating evidence demonstrates that inflammatory pathways play a critical role in the pathogenesis of obesity-related metabolic disorders. In particular, white adipose tissue (WAT) from individuals with obesity exhibits a pronounced inflammatory phenotype, characterized by extensive infiltration of immune cells, including macrophages, lymphocytes, and dendritic cells [2,3]. This chronic inflammatory microenvironment within WAT contributes to metabolic dysregulation and the development of insulin resistance. Myeloid differentiation factor 88 (MyD88) is a central adaptor protein for Toll-like receptor (TLR) and interleukin-1 receptor (IL-1R) signaling and is a key mediator of inflammatory pathway activation. Activation of the TLR4/MyD88/NF-κB axis stimulates the production of proinflammatory cytokines such as tumor necrosis factor-α (TNF-α), interleukin-6 (IL-6), and monocyte chemoattractant protein-1 (MCP-1), thereby promoting metabolic inflammation and T2DM-related complications [4]. In patients with T2DM, elevated expression of phosphorylated IL-1R-associated kinase 1, TLR4, MyD88, and downstream inflammatory mediators has been detected in circulating monocytes, further supporting the involvement of MyD88-dependent inflammatory signaling in diabetes pathogenesis [5]. However, studies in high-fat-diet-fed MyD88-deficient mice have reported paradoxical metabolic disturbances, including hyperinsulinemia, dyslipidemia, hyperleptinemia, and liver dysfunction, suggesting a complex and context-dependent role of MyD88 in metabolic regulation [6]. Thus, the precise functions of MyD88 signaling in adipose tissue inflammation and diabetes-associated metabolic complications remain incompletely understood.

Interleukin-10 (IL-10) is a pleiotropic anti-inflammatory cytokine produced by multiple immune cell types. Myeloid dendritic cells and macrophages express IL-10 upon activation of MyD88- and TRIF-dependent TLR pathways, including TLR3 and TLR4, in response to double-stranded RNA and lipopolysaccharide (LPS), respectively [7]. Endogenous IL-10 has been shown to exert protective effects against diet-induced hepatic insulin resistance [8]. Conversely, suppression of IL-10 enhances proinflammatory cytokine expression, exacerbates insulin signaling defects, and activates gluconeogenic and lipogenic pathways [8]. Notably, adipose tissue macrophage-derived IL-10, induced by insulin and LPS, suppresses hepatic glucose production in coordination with insulin signaling [9]. These findings suggest that dysregulation of IL-10 production in adipose tissue may represent a critical mechanistic link between inflammation and hepatic gluconeogenesis in T2DM.

Regulatory T cells (Tregs), defined by the expression of the transcription factor forkhead box P3 (Foxp3), play a pivotal role in maintaining immune homeostasis and preventing excessive inflammatory responses [10]. A substantial population of Tregs resides within adipose tissue, where they contribute to the suppression of local inflammation and preservation of metabolic homeostasis [10]. However, whether MyD88-dependent inflammatory signaling modulates adipose Treg homeostasis and IL-10 production to regulate hepatic inflammation and gluconeogenesis in diabetes remains unclear. In this study, we investigate the role of MyD88 signaling in regulating adipose IL-10 production, Treg homeostasis, hepatic inflammation, and gluconeogenesis in diabetes mellitus. We hypothesized that MyD88 activation promotes hepatic inflammation and gluconeogenesis by suppressing adipose-derived IL-10 and Foxp3-expressing Tregs in T2DM. To test this hypothesis, we used MyD88 deficiency in a leptin receptor gene mutation mouse model and examined adipose immune responses, hepatic inflammatory signaling, insulin resistance, and gluconeogenic gene expression in a diabetic context. In addition, we administered IL-10 directly into adipose tissue and evaluated its effects on inflammatory gene expression, Treg abundance, circulating dipeptidyl peptidase-4 activity, adiponectin levels, hepatic Akt activation, gluconeogenic gene expression, and glucose tolerance in a T2DM mouse model. Our findings provide mechanistic insight into the MyD88/IL-10 axis in adipose–liver crosstalk and suggest that targeting this pathway may represent a novel therapeutic strategy for metabolic syndrome.

## 2. Results

### 2.1. Lepr^db/db^MyD88^−/−^ Mice Exhibited Increased IL-10 Levels and Foxp3 Expression in Adipose Tissue SVFs and Reduced Circulating Adiponectin and DPP4 Activity

**Figure 1:**
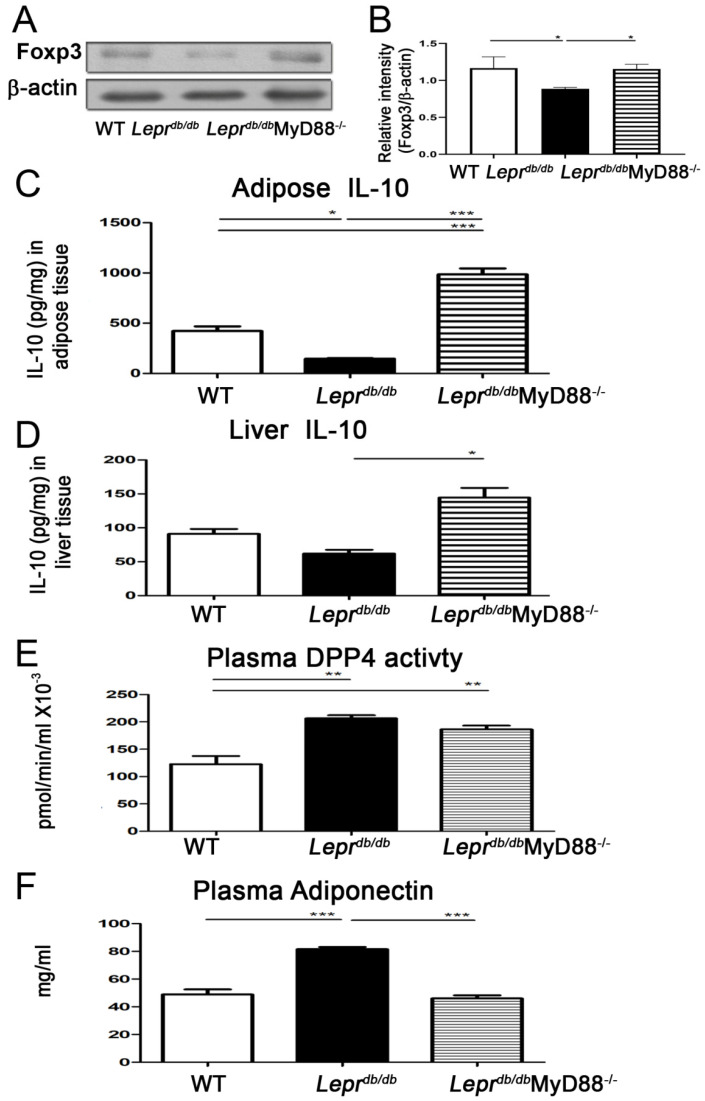
MyD88 deficiency in *Lepr^db^*^/*db*^ mice enhances IL−10 in adipose tissue and liver and decreased circulating adiponectin and DPP4 activity. Adipose tissue, liver, and blood were collected from *Lepr^+/+^*, *Lepr^db^*^/*db*^, and *Lepr^db^*^/*db*^MyD88^−/−^ mice to examine the effects of MyD88 signaling deletion. (**A**) Foxp3 protein expression in stromal vascular fractions (SVFs) isolated from adipose tissue was assessed by Western blotting. (**B**) Densitometric quantification of Foxp3 expression illustrated in (**A**). (**C**,**D**) IL−10 protein levels in adipose tissue (**C**) and liver (**D**) were measured by ELISA. (**E**) Plasma dipeptidyl peptidase-4 (DPP4) enzymatic activity was determined using a commercial DPP4 activity assay kit (BioVision, Milpitas, CA, USA, # K779-100). (**F**) Circulating adiponectin levels were quantified by ELISA. Data are presented as mean ± SEM. *n* = 5 mice per group. * *p* < 0.05, ** *p* < 0.01, *** *p* < 0.001. Foxp3, forkhead box P3.

Adipose tissue, liver, and blood were collected from *Lepr*^+/+^, *Lepr^db^*^/*db*^, and *Lepr^db^*^/*db*^MyD88^−/−^ mice to examine the effects of MyD88 deficiency on FOXp3 expression in stromal vascular fractions (SVFs), IL-10 levels in adipose tissue and liver, and circulating adiponectin levels and DPP4 activity. SVFs from *Lepr^db^*^/*db*^ mice showed significantly reduced Foxp3 protein expression compared with *Lepr*^+/+^ mice (Figure 1A,B and Figure S1). In addition, *Lepr^db^*^/*db*^ mice exhibited decreased IL-10 levels in adipose tissue and increased plasma DPP4 activity and adiponectin levels (Figure 1C–F). In contrast, *Lepr^db^*^/*db*^MyD88^−/−^ mice demonstrated significantly increased Foxp3 expression in SVFs and elevated IL-10 levels in both adipose tissue and liver compared with *Lepr^db^*^/*db*^ mice (Figure 1A–D). Moreover, MyD88 deficiency significantly reduced circulating adiponectin levels in diabetic mice (Figure 1F). These results indicate that T2DM suppresses adipose Foxp3 and IL-10 expression while increasing plasma DPP4 activity and adiponectin levels, and that MyD88 deletion reverses these alterations.

### 2.2. Lepr^db/db^MyD88^−/−^ Mice Displayed Reduced Hepatic Inflammatory and Gluconeogenic Gene Expression

**Figure 2:**
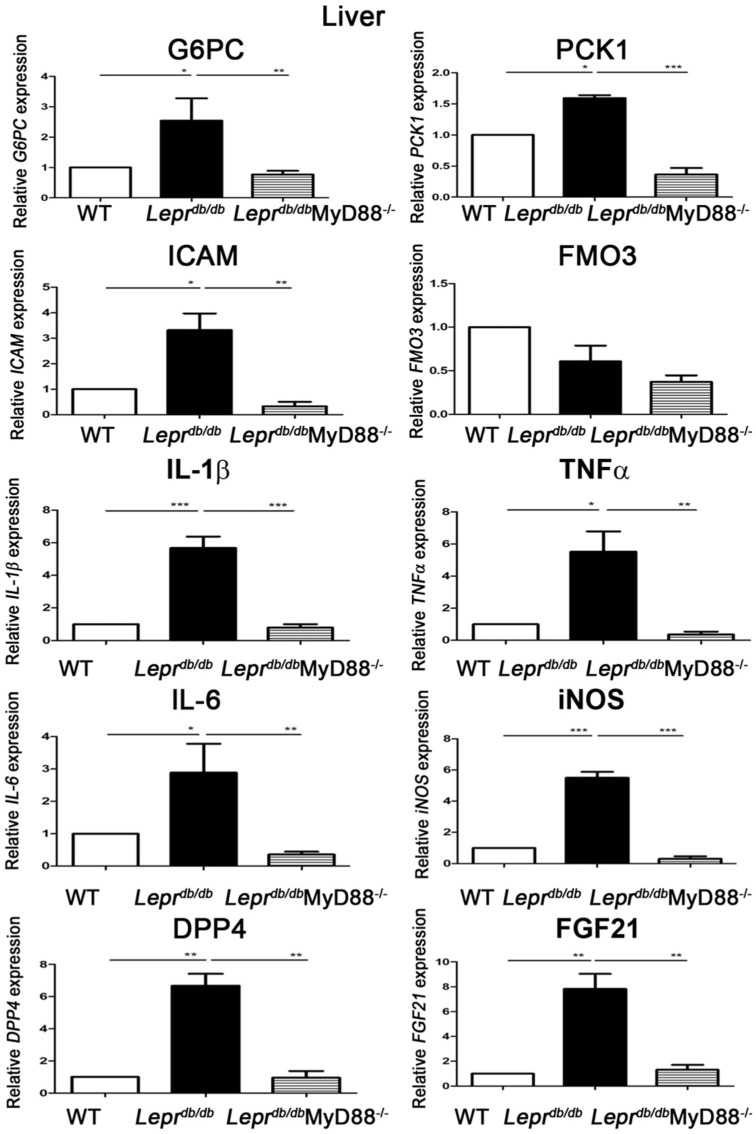
MyD88 depletion suppresses hepatic inflammatory and gluconeogenic gene expression in *Lepr^db^*^/*db*^ mice. Liver tissues from *Lepr*^+/+^, *Lepr^db^*^/*db*^, and *Lepr^db^*^/*db*^MyD88^−/−^ mice were harvested for quantitative PCR (Q-PCR) analysis. Hepatic mRNA expression levels of inflammatory markers (*ICAM*, *IL*−*1β*, *TNF*−*α*, *IL*−*6*, *iNOS*), metabolic regulators (*DPP4*, *FGF21*), and gluconeogenic enzymes (*G6pc* and *Pck1*) were determined. Data are presented as mean ± SEM. * *p* < 0.05, ** *p* < 0.01, *** *p* < 0.001.

To evaluate hepatic inflammatory and metabolic gene regulation, liver tissues were analyzed by Q-PCR. *Lepr^db^*^/*db*^ mice exhibited significantly elevated mRNA expression of *ICAM*, *IL-1β*, *TNF-α*, *IL-6*, *iNOS*, *DPP4*, *FGF21*, *G6pc*, and *Pck1* compared with *Lepr*^+/+^ mice (Figure 2). In contrast, *Lepr^db^*^/*db*^MyD88^−/−^ mice showed marked reductions in all of these transcripts relative to *Lepr^db^*^/*db*^ mice (Figure 2). These findings indicate that MyD88 signaling is a critical contributor to hepatic inflammation and gluconeogenesis in T2DM.

### 2.3. MyD88 Deficiency Reduced Inflammatory Gene Expression in Kupffer Cells and Improved Liver Function

**Figure 3:**
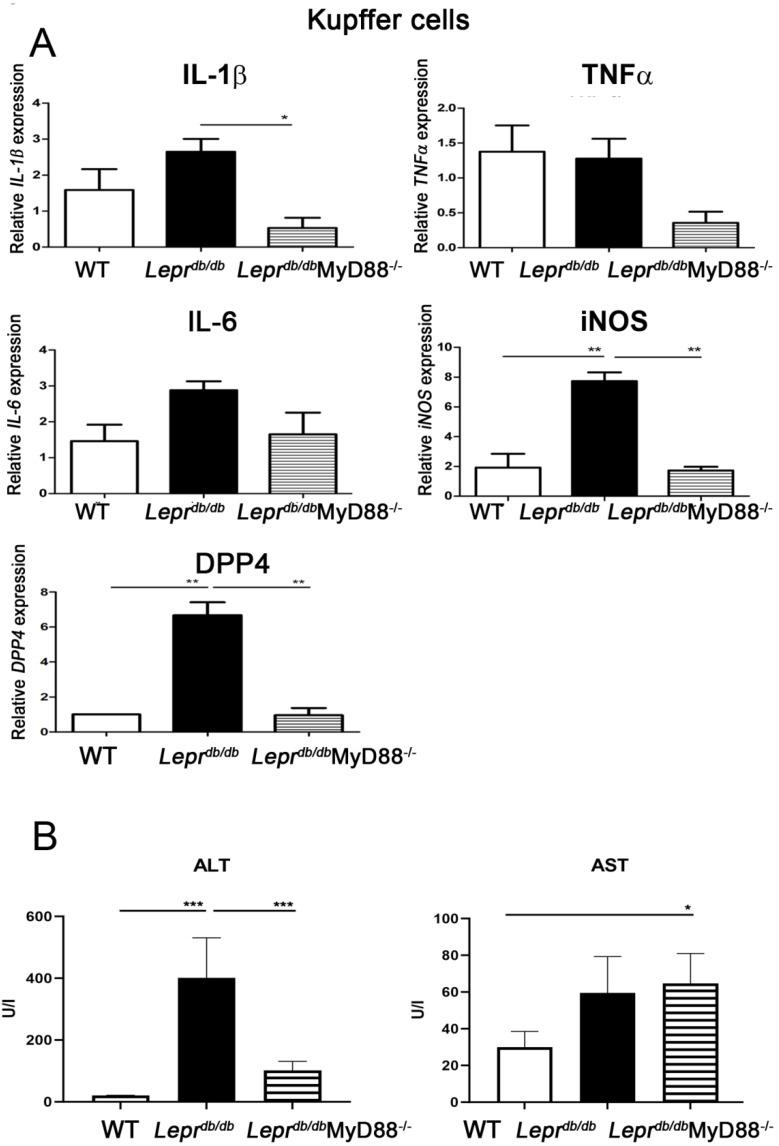
MyD88 deficiency reduces inflammatory gene expression in Kupffer cells and attenuates liver injury in *Lepr^db^*^/*db*^ mice. Kupffer cells and blood samples were isolated from *Lepr*^+/+^, *Lepr^db^*^/*db*^, and *Lepr^db^*^/*db*^MyD88^−/−^ mice. (**A**) mRNA expression of inflammatory mediators (*IL*−*1β*, *TNF*−*α*, *IL*−*6*, *iNOS*, and *DPP4*) in Kupffer cells was quantified by Q-PCR. (**B**) Serum alanine aminotransferase (ALT) levels were measured as an indicator of hepatic injury. Data are presented as mean ± SEM. *n* = 5 mice per group. * *p* < 0.05, ** *p* < 0.01, *** *p* < 0.001. ALT alanine aminotransferase, AST aspartate transaminase.

Kupffer cells isolated from *Lepr^db^*^/*db*^ mice demonstrated significantly increased iNOS and DPP4 mRNA expression compared with *Lepr*^+/+^ mice (Figure 3A). MyD88 deletion significantly reduced *IL-1β*, iNOS, and DPP4 expression in Kupffer cells relative to *Lepr^db^*^/*db*^ mice. Consistently, serum ALT levels were elevated in *Lepr^db^*^/*db*^ mice and significantly reduced in *Lepr^db^*^/*db*^MyD88^−/−^ mice (Figure 3B), indicating improved liver function.

### 2.4. SVFs from Lepr^db/db^MyD88^−/−^ Mice Exhibited Reduced Inflammatory Cytokines and Increased PDGFα Expression and IL-10 Treatment Recapitulated These Effects

**Figure 4:**
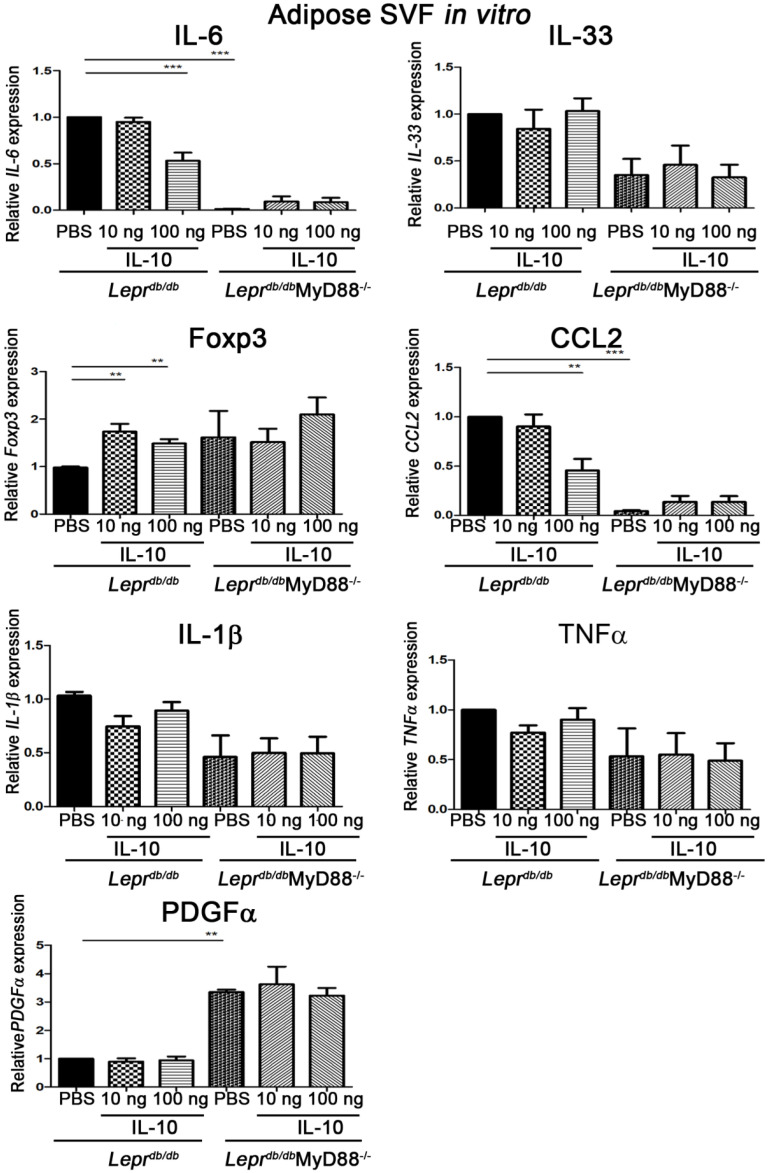
Altered cytokine expression in adipose SVFs from *Lepr^db/db^*MyD88^−/−^ mice and modulation by IL−10 treatment SVFs were harvested from the adipose tissue of *Lepr^db/db^* and *Lepr^db/db^*MyD88^−/−^ mice and purified for mRNA expression of different cytokines by Q-PCR analysis to examine *IL-6*, *IL*−*33*, *Foxp3*, *CCL2*, *IL*−*1β*, *TNF*−*α*, and *PDGFα* cytokine mRNA expression in adipose SVFs and MyD88 involvement. Furthermore, SVFs (2 × 10^7^ cells) purified from the adipose tissue of *Lepr^db/db^* and *Lepr^db/db^*MyD88^−/−^ mice were treated with PBS or 10 and 100 ng of IL−10 for 3.5 h followed by Q-PCR analysis of *IL*−*6*, *IL*−*33*, *Foxp3*, *CCL2*, *IL*−*1β*, *TNF*−*α*, and *PDGFα* mRNA expression. *n* = 5/group. ** *p* < 0.01, *** *p* < 0.001. SVF, stromal vascular fraction; forkhead box p3, *Foxp3; PDGF, platelet-derived growth factor.*

SVFs from *Lepr^db^*^/*db*^MyD88^−/−^ mice displayed significantly reduced *IL-6* and *CCL2* expression and increased *PDGFα* mRNA levels compared with *Lepr^db^*^/*db*^ mice (Figure 4). In vitro IL-10 treatment of SVFs from *Lepr^db^*^/*db*^ mice significantly increased Foxp3 expression at 10 ng and reduced *IL-6* and *CCL2* while augmenting Foxp3 at 100 ng (Figure 4). In contrast, IL-10 treatment did not further alter cytokine expression in SVFs from *Lepr^db^*^/*db*^MyD88^−/−^ mice. These findings indicate that MyD88 signaling mediates proinflammatory cytokine expression in adipose SVFs and that IL-10 suppresses inflammation and promotes Foxp3 expression via a MyD88-dependent mechanism.

### 2.5. IL-10 Injection Enhanced Foxp3 and IL-10 Expression and Suppressed JNK and NF-κB Signaling in Adipose SVFs

**Figure 5:**
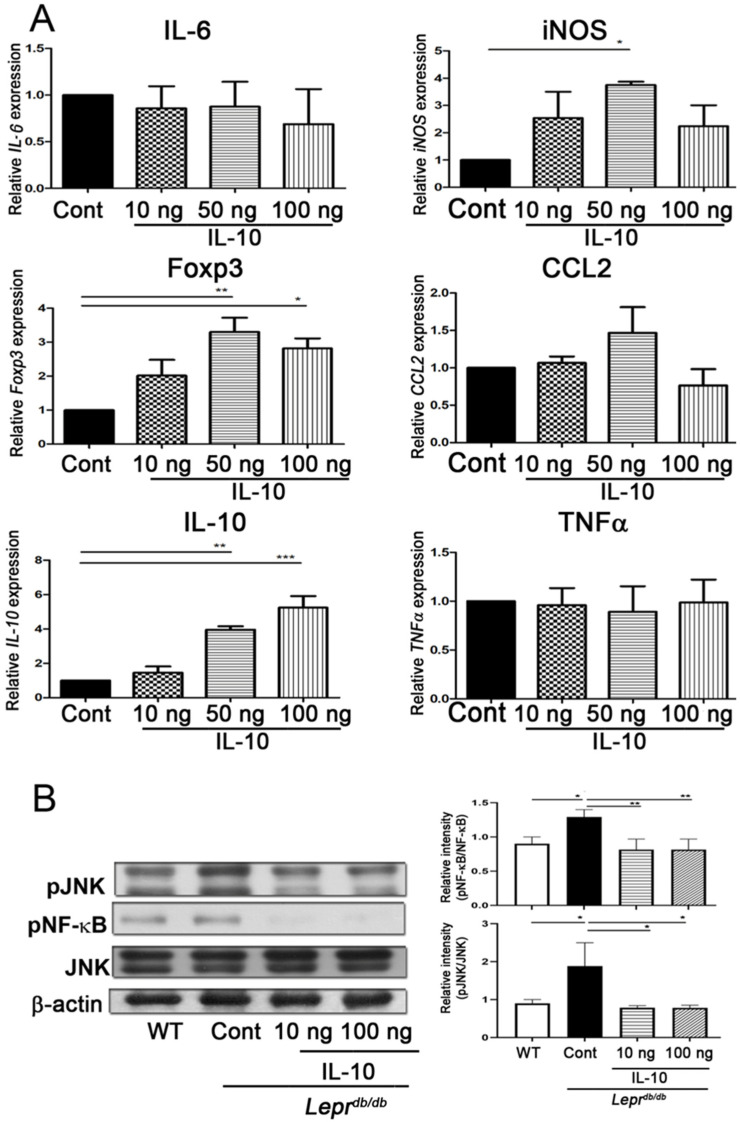
Local IL−10 administration induces *Foxp3* expression and suppresses inflammatory signaling pathways in adipose SVFs of *Lepr^db/db^* mice. PBS or IL−10 (10, 50, or 100 ng) was injected directly into adipose tissue of *Lepr^db/db^* mice. SVFs were isolated 7 days after injection. (**A**) mRNA expression of inflammatory cytokines (*IL*−*6*, *IL-33*, *CCL2*, *IL*−*1β*, *TNF*−*α*) and *Foxp3* was quantified by Q-PCR. (**B**) Protein expression of phosphorylated and total JNK and NF-κB was evaluated by Western blotting to assess inflammatory signaling activity. Data are presented as mean ± SEM. *n* = 5 mice per group. * *p* < 0.05, ** *p* < 0.01, *** *p* < 0.001.

In vivo injection of IL-10 into adipose tissue of *Lepr^db/db^* mice significantly increased *Foxp3* and *IL-10* mRNA expression at doses of 50 and 100 ng (Figure 5A). Moreover, 10 ng IL-10 significantly reduced pJNK and pNF-κB protein levels in SVFs (Figure 5B and Figure S2). These results demonstrate that IL-10 suppresses inflammatory signaling pathways in diabetic adipose tissue.

### 2.6. IL-10 Promoted CD4^+^ Regulatory T-Cell Accumulation in Adipose Tissue

**Figure 6:**
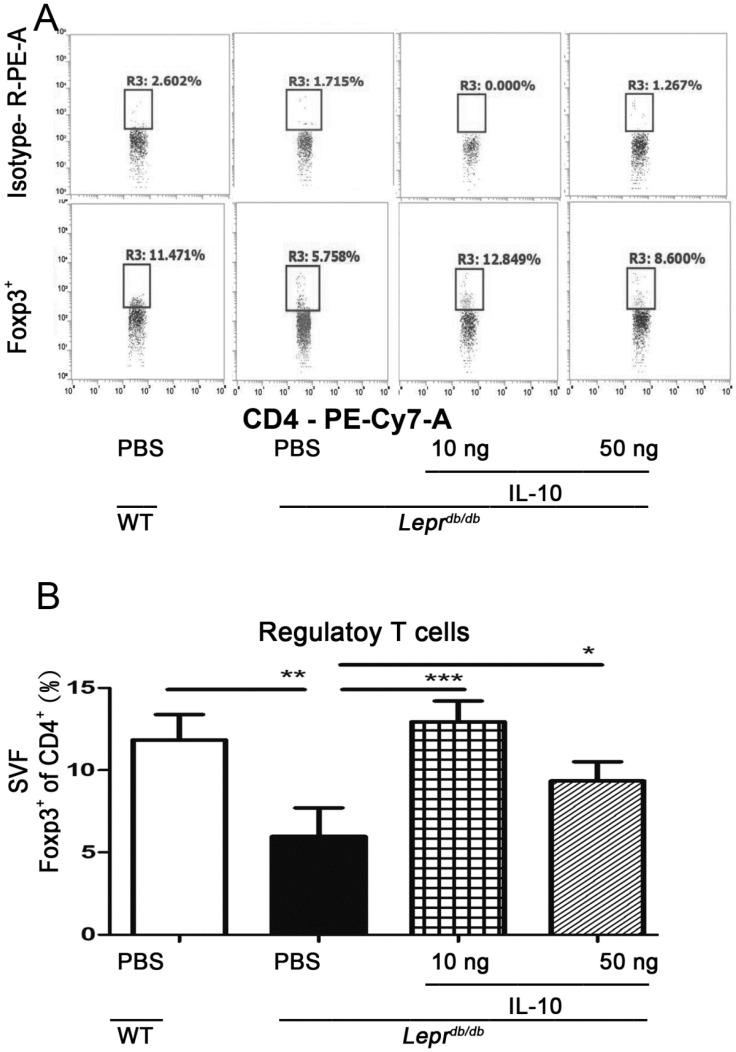
IL−10 injection increases regulatory T cell accumulation in adipose tissue of *Lepr^db/db^* mice. PBS or IL−10 (10, 50, or 100 ng) was injected into adipose tissue of *Lepr^db/db^* mice. SVFs were isolated 7 days after injection and analyzed by flow cytometry. (**A**) Representative flow cytometry plots showing CD4^+^ regulatory T cells (Tregs). (**B**) Quantification of the frequency and absolute number of CD4^+^ Tregs in adipose tissue. Data are presented as mean ± SEM. N = 5 mice per group. * *p* < 0.05, ** *p* < 0.01, *** *p* < 0.001.

IL-10 induction promotes Treg differentiation [11]. Flow cytometry analysis revealed a significant increase in CD4^+^ Foxp3^+^ Tregs in adipose tissue of *Lepr^db/db^* mice receiving IL-10 (10 or 50 ng) compared with PBS-treated controls (Figure 6A,B). This effect indicates that IL-10 administration increases CD4^+^ Tregs in SVFs from the adipose tissue of T2DM.

### 2.7. IL-10 Injection Reduced Circulating Adiponectin and DPP4 Activity

**Figure 7:**
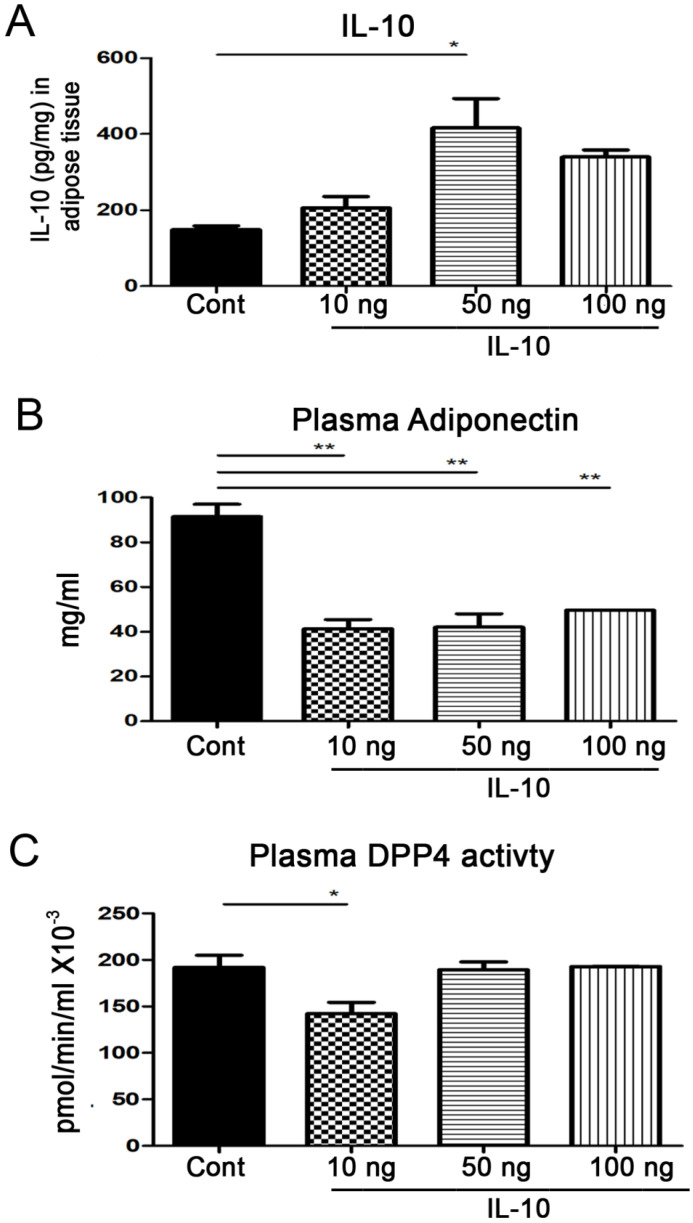
IL−10 administration decreased blood adiponectin levels and DPP4 activity in *Lepr^db/db^* mice. PBS or IL−10 (10, 50, or 100 ng) was injected into adipose tissue of *Lepr^db/db^* mice, and tissues were harvested 7 days later. (**A**) IL-10 protein levels in adipose tissue were measured by ELISA. (**B**) Plasma adiponectin levels were determined by ELISA. (**C**) Plasma DPP4 enzymatic activity was assessed using a DPP4 activity assay kit (BioVision, Milpitas, CA, USA, # K779-100). Data are presented as mean ± SEM. *n* = 5 mice per group. * *p* < 0.05, ** *p* < 0.01.

Injection of 50 ng IL-10 significantly increased adipose IL-10 levels (Figure 7A). IL-10 administration at 10, 50, and 100 ng significantly reduced circulating adiponectin levels compared with PBS controls (41.34 ± 4.22, 42.12 ± 5.99, or 49.72 ± 6.12 vs. 91.47 ± 5.56 pg/mL) (Figure 7B). In addition, 10 ng IL-10 significantly lowered plasma DPP4 activity (148.3 ± 10.59 vs. 193.1 ± 9.11 pmol/min/mL × 10^−3^) (Figure 7C), indicating systemic metabolic improvement.

### 2.8. IL-10 Reduced ICAM, TNF-α, IL-6, DPP4, and iNOS mRNA Expression and Increased pAkt and pERK Levels in the Liver

**Figure 8:**
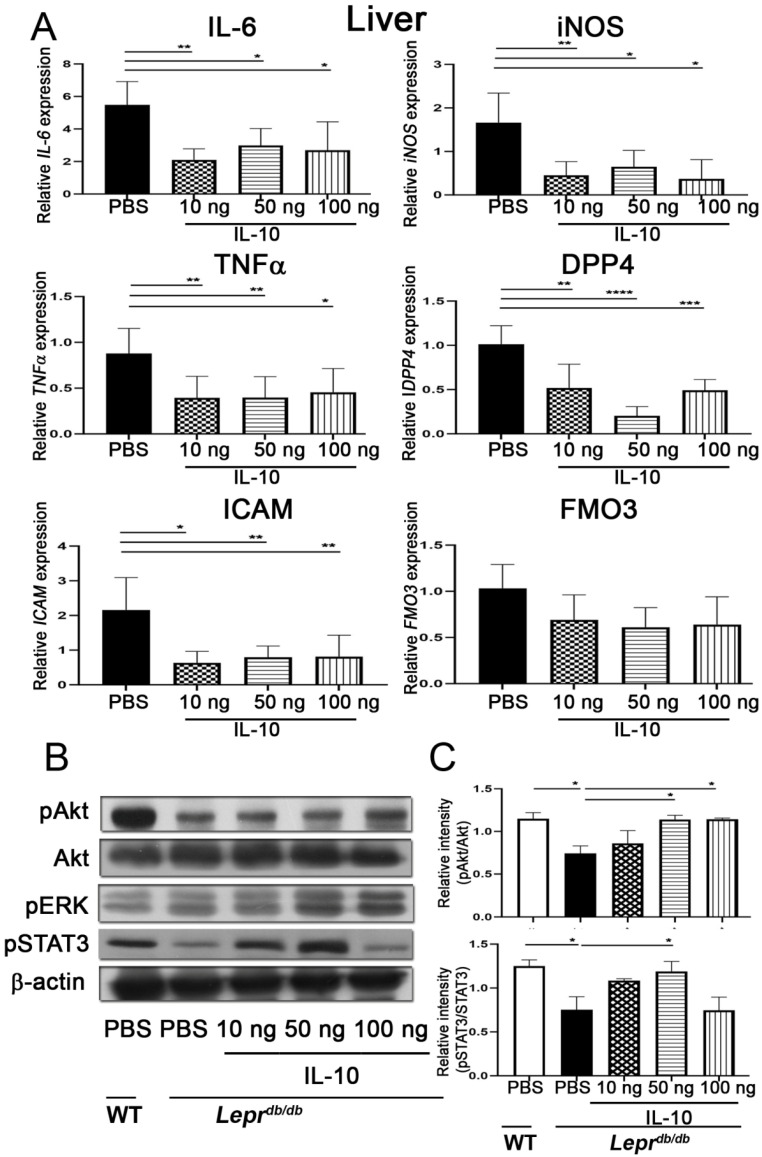
IL−10 administration suppresses hepatic *ICAM*, *TNF*−*α*, *IL*−*6*, *DPP4*, and *iNOS* mRNA expression and activates insulin-related signaling pathways in *Lepr^db/db^* mice. PBS or IL-10 (10, 50, or 100 ng) was injected into adipose tissue of *Lepr^db/db^* mice, and livers were harvested 7 days later. (**A**) Hepatic mRNA expression of *ICAM*, *FGF21*, *IL*−*1β*, *TNF*−*α*, *DPP4*, and *iNOS* was determined by Q-PCR. (**B**) Protein expression of phosphorylated and total Akt, STAT3, and ERK was assessed by Western blotting. (**C**) Quantification of the pAkt/Akt and pSTAT3/STAT3 ratio. Data are presented as mean ± SEM. *n* = 4 mice per group. * *p* < 0.05, ** *p* < 0.01, *** *p* < 0.001, **** *p* < 0.0001.

PBS or 10, 50, and 100 ng of IL-10 were injected into adipose tissue of T2DM (*Lepr^db/db^*) mice, and the liver was harvested 7 days after injection to further examine whether IL-10 injection decreased inflammatory cytokines mRNA expression of the liver. IL-10 injection at 10, 50, or 100 ng significantly decreased *ICAM*, *TNF-α*, *IL-6*, *DPP4*, and *iNOS* mRNA expression of the liver in *Lepr^db/db^* mice compared with PBS injection (Figure 8A). T2DM (*Lepr^db/db^*) mice demonstrated a significant decrease in pAkt protein and pSTAT3 protein expression in the liver compared with *Lepr*^+/+^ mice. IL-10 injection at 100 ng into adipose tissue of T2DM (*Lepr^db/db^*) mice significantly increased pAkt and pERK protein expression in the liver compared with PBS injection group (Figure 8B,C and Figure S3). Collectively, our results reveal that IL-10 injection into adipose tissue reduces inflammatory cytokines mRNA expression and increases pAkt and pERK levels in the liver of a T2DM mouse model.

### 2.9. IL-10 Reduced Inflammatory Gene Expression in Kupffer Cells and Improved Liver Function

**Figure 9:**
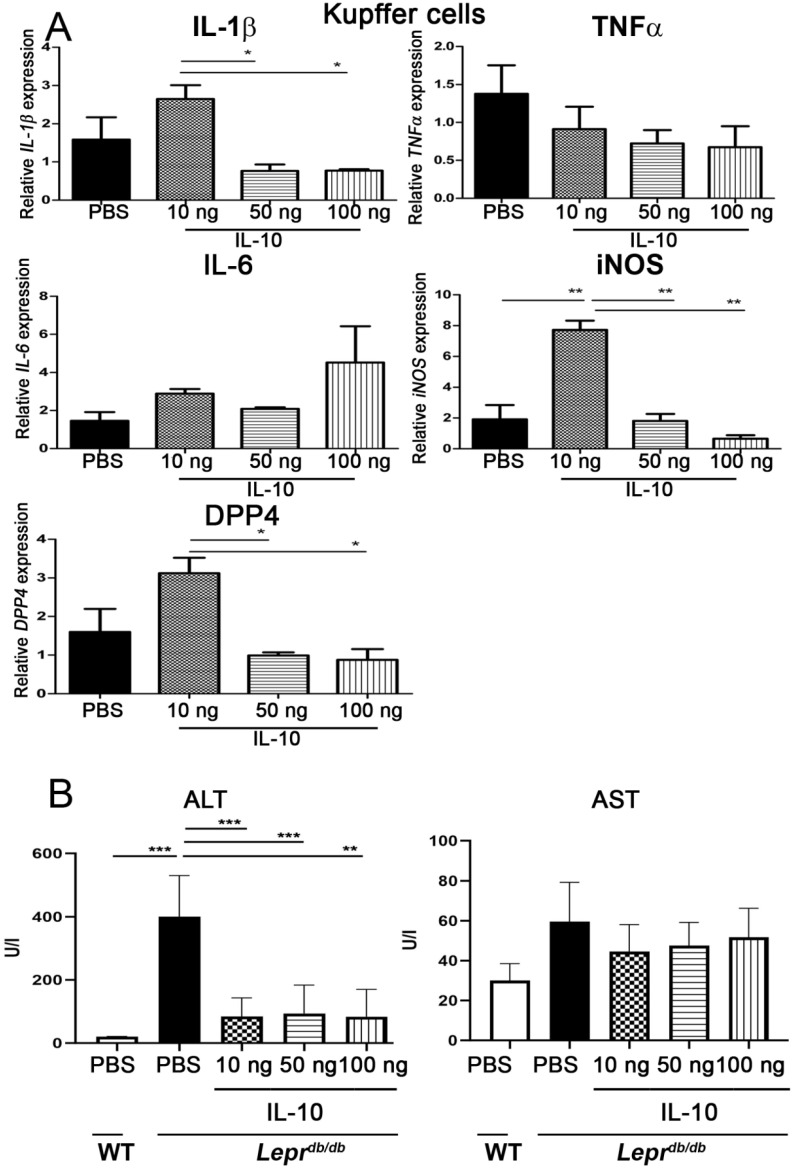
IL−10 injection reduces inflammatory gene expression in Kupffer cells and improves liver injury markers in *Lepr^db/db^* mice. PBS or IL−10 (10, 50, or 100 ng) was injected into adipose tissue of *Lepr^db/db^* mice. Kupffer cells and blood were collected 7 days after injection. (**A**) Kupffer cell mRNA expression of *IL-*−*1β*, *TNF*−*α*, *IL*−*6*, *iNOS*, and *DPP4* was quantified by Q-PCR analysis. (**B**) Serum ALT levels were measured to assess hepatic injury. Data are presented as mean ± SEM. *n* = 6 mice per group. * *p* < 0.05, ** *p* < 0.01, *** *p* < 0.001. ALT alanine aminotransferase, AST aspartate transaminase.

IL-10 administration significantly reduced IL-1β, iNOS, and DPP4 mRNA expression in Kupffer cells at 10 and 50 ng compared with PBS (Figure 9A). Serum ALT levels were significantly reduced at all IL-10 doses (Figure 9B), demonstrating improved hepatic function.

### 2.10. IL-10 Reduced Hepatic Gluconeogenic Gene Expression

**Figure 10:**
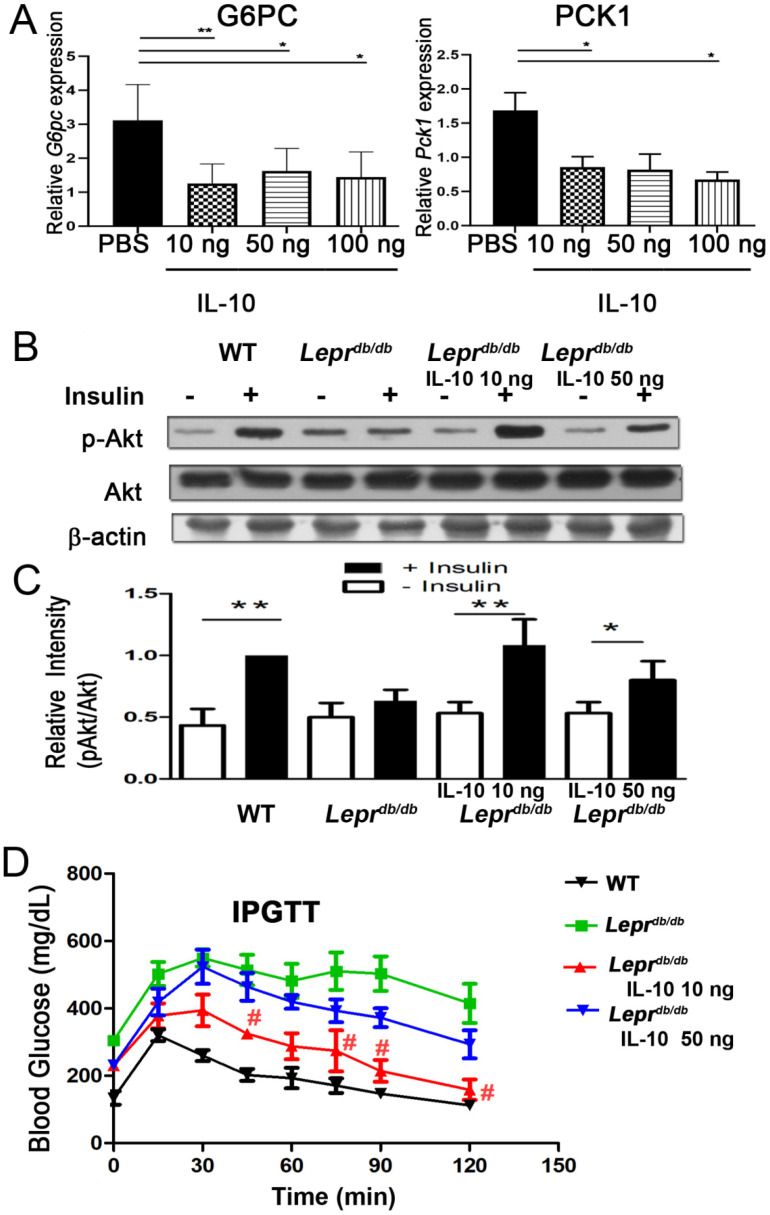
IL−10 administration reduces hepatic gluconeogenic gene expression, enhances Akt activation, and improves glucose tolerance in *Lepr^db/db^* mice. PBS or IL-10 (10 or 50 ng) was injected into inguinal white adipose tissue (WAT) of *Lepr^db/db^* mice, the liver was harvested 7 days after injection and subjected to Q-PCR analysis to determine the expression of *G6pc* and *Pck1* mRNA (**A**). (**B**) One week after injection, mice were treated with insulin (1.25 mU/g body weight) for 20 min, followed by isolation of SVFs and Western blot analysis of phosphorylated and total Akt. (**C**) Quantification of the pAkt/Akt ratio. (**D**) Glucose tolerance tests were performed by intraperitoneal glucose administration (1 g/kg body weight), with blood glucose measured at baseline and at 15 min intervals for 2 h. Data are presented as mean ± SEM. *n* = 4 mice per group. * *p* < 0.05, ** *p* < 0.01; # *p* < 0.05 compared with PBS-treated *Lepr^db/db^* mice.

PBS or 10, 50, and 100 ng of IL-10 were injected into adipose tissue of T2DM (*Lepr^db/db^*) mice, and the liver was harvested 7 days after injection to further examine whether IL-10 injection decreased gluconeogenesis-related gene mRNA expression of the liver. IL-10 injection at 50 ng significantly reduced hepatic *G6pc* and *Pck1* mRNA expression (Figure 10A). Collectively, our results reveal that IL-10 injection into adipose tissue reduces hepatic *G6pc* and *Pck1* mRNA expression.

### 2.11. IL-10 Enhanced Insulin Sensitivity and Improved Glucose Tolerance in Diabetic Mice

*Lepr^db/db^* mice showed impaired hepatic Akt phosphorylation after insulin stimulation compared with *Lepr*^+/+^ mice (Figure 10B,C and Figure S4). IL-10 treatment at 10 ng restored insulin-induced Akt activation. During glucose tolerance testing, *Lepr^db/db^* mice displayed sustained hyperglycemia, whereas 10 ng IL-10 significantly improved glucose clearance. The 50 ng dose produced only modest improvement (Figure 10D). These findings indicate that adipose IL-10 enhances systemic insulin sensitivity and glucose tolerance in T2DM.

## 3. Discussion

Previous studies have demonstrated that activation of the TLR4/MyD88/NF-κB signaling pathway induces the production of proinflammatory cytokines, including TNF-α, IL-6, IL-8, and MCP-1, thereby contributing to cardiovascular and hepatic complications in T2DM models [4]. However, paradoxically, MyD88-deficient mice subjected to a high-fat diet have also been reported to develop hyperinsulinemia, hyperleptinemia, hypercholesterolemia, and liver dysfunction, suggesting a context-dependent role of MyD88 in metabolic regulation [6]. In the present study, we aimed to clarify the role of MyD88 signaling in diabetes-associated hepatic inflammation and gluconeogenesis using *Lepr^db/db^*MyD88^−/−^ mice. Our findings demonstrate that T2DM induces adipose and hepatic inflammation, elevates circulating ALT and DPP4 activity, and suppresses adipose Foxp3 expression and IL-10 production in a MyD88-dependent manner. Importantly, genetic deletion of MyD88 markedly reversed these pathological alterations, indicating that MyD88 signaling is a central regulator of metabolic inflammation in T2DM.

We first demonstrated that MyD88 signaling plays a critical role in mediating diabetes-induced adipose and hepatic inflammation. *Lepr^db/db^* mice exhibited significantly reduced Foxp3 expression in SVFs and decreased IL-10 levels in adipose tissue, accompanied by elevations in circulating DPP4 activity and adiponectin levels. In contrast, *Lepr^db/db^*MyD88^−/−^ mice showed restoration of IL-10 expression in adipose tissue and liver and a reduction in circulating adiponectin levels. Furthermore, hepatic expression of inflammatory and metabolic genes, including *ICAM*, *IL-1β*, *TNF-α*, *IL-6*, *iNOS*, *DPP4*, *FGF21*, *G6pc*, and *Pck1*, as well as inflammatory markers in Kupffer cells, was markedly increased in *Lepr^db/db^* mice and significantly attenuated by MyD88 deletion. These findings collectively indicate that MyD88 signaling is required for the development of adipose and hepatic inflammation, as well as liver dysfunction, in T2DM.

IL-10 is a key immunoregulatory cytokine that suppresses excessive inflammatory responses and promotes immune tolerance [12]. In the presence of TGF-β, IL-10 facilitates the expansion of Foxp3^+^ regulatory T cells with enhanced *CTLA-4* (cytotoxic T-lymphocyte-associated protein 4) expression [11]. The mechanisms proposed for T1DM regulation by the involved IL-10 increase in Treg frequencies and Th2-type cytokine (IL-4 and IL-10) levels and IL-2 and IFN-c cytokine suppression [13]. Our data demonstrate that MyD88 deficiency significantly increased IL-10 production in adipose tissue and liver and concurrently reduced hepatic inflammation and gluconeogenic gene expression in T2DM. Moreover, MyD88 activation promoted adipose *IL-6* and *CCL2* expression, hepatic inflammation, gluconeogenesis, and circulating adiponectin levels by suppressing IL-10 and Foxp3 in adipose tissue. Consistent with this mechanism, SVFs from *Lepr^db/db^*MyD88^−/−^ mice displayed reduced expression of *IL-6* and *CCL2* and increased *PDGFα* expression. In vitro IL-10 treatment of SVFs from *Lepr^db/db^* mice decreased *IL-6* and *CCL2* while increasing *Foxp3* expression. In vivo administration of IL-10 further confirmed these observations by reducing pJNK and pNF-κB signaling and enhancing Foxp3 and IL-10 expression in adipose SVFs. These results indicate that IL-10 suppresses MyD88-driven inflammatory signaling and promotes Treg-associated immune regulation in adipose tissue under diabetic conditions.

Importantly, IL-10 administration also exerted profound metabolic benefits. IL-10 injection significantly reduced hepatic *G6pc* and *Pck1* expression, decreased inflammatory gene expression in Kupffer cells, lowered serum ALT levels, and restored insulin-stimulated Akt activation in the liver. Furthermore, low-dose IL-10 (10 ng) significantly improved glucose tolerance and reduced circulating DPP4 activity. These findings indicate that adipose-derived IL-10 not only suppresses local inflammation but also improves systemic insulin sensitivity and hepatic glucose metabolism. The observation that low-dose IL-10 was more effective than higher doses in improving insulin sensitivity and glucose tolerance suggests a dose-dependent and tightly regulated role of IL-10 in metabolic control. A previous study showed that IL-10 treatment can upregulate phosphorylated Akt (pAkt) to mediate anti-apoptotic, pro-survival, and anti-inflammatory effects, particularly by activating the PI3K/Akt pathway alongside JAK1/STAT3 signaling [14]. DPP4, Tregs, and IL-10 are closely interconnected in the regulation of inflammation and immune tolerance. Previous studies have shown that DPP4 inhibition enhances Treg proliferation and IL-10 production, contributing to anti-inflammatory effects [15]. DPP4 inhibitors, widely used in the treatment of T2DM, have also been reported to exert immunomodulatory properties through IL-10 induction [16]. Recently, chimeric antigen receptor (CAR) cell therapy has been explored as a novel therapeutic approach in alleviating liver fibrosis and may become a future therapeutic strategy for hepatic metabolic syndrome [17]. Our previous work demonstrated that M1/M2 macrophage polarization in adipose tissue critically regulates diabetes-associated DPP4 activity, hepatic inflammation, and insulin resistance [18]. The present study extends these findings by identifying MyD88 as a key upstream regulator of the adipose IL-10–Treg–DPP4 axis in T2DM.

PDGF acts as pro-inflammatory mediators, contributing to remodeling, pain, and fibrosis in conditions such as arthritis, asthma, and fibrotic diseases. However, PDGF can induce tolerogenic effects, such as inducing IL-10 in dendritic cells and decreasing TNF-α, suggesting a complex and dual role [19]. Adiponectin is an adipokine with well-established protective roles in metabolic homeostasis [20], inflammation, and atherosclerosis [21,22]. Unlike leptin, adiponectin levels are typically reduced in obesity and insulin resistance [3]. Therefore, we examined plasma adiponectin levels in our model. Interestingly, our data revealed significantly decreased adiponectin levels and increased *PDGFα* expression in adipose SVFs of *Lepr^db/db^*MyD88^−/−^ mice compared with *Lepr^db/db^* mice. Moreover, IL-10 injection significantly reduced circulating adiponectin levels in diabetic mice. These findings suggest that MyD88 signaling suppresses *IL-10* and *PDGFα* expression while promoting adiponectin production in T2DM. Although this result appears paradoxical given the traditionally protective role of adiponectin, it highlights the complex and context-dependent regulation of adipokines under chronic inflammatory conditions.

Our study has several limitations. We did not directly compare the effects of IL-10 administration in IL-10 receptor-deficient mice or perform adoptive transfer of IL-10^+^Foxp3^+^ Tregs to definitively establish causality between IL-10, Treg expansion, and hepatic STAT3 signaling. Future studies using IL-10 receptor knockout models and Treg adoptive transfer approaches will be required to further delineate the precise cellular and molecular mechanisms involved.

## 4. Materials and Methods

### 4.1. Animals

*Lepr^db/+^* mice were obtained from The Jackson Laboratory (Bar Harbor, ME, USA) and bred to generate diabetic *Lepr^db/db^* and non-diabetic *Lepr*^+/+^ mice littermates. *Lepr^db/db^* mice carry a mutation in the leptin receptor gene and develop obesity at 3 to 4 weeks of age, with hyperinsulinemia and hyperglycemia evident between 4 and 8 weeks. We generated *Lepr^db/db^*MyD88^−/−^ mice were generated by crossbreeding *Lepr^db/db^* mice with MyD88^−/−^ mice.

All mice (*Lepr*^+/+^, *Lepr^db/db^*, *Lepr^db/db^*MyD88^−/−^) were maintained on a standard laboratory diet (1324 TPF; Atromin; Lage, Germany; 11.9 kJ/g, 19% crude protein, 4% crude fat, 6% crude fiber) with ad libitum access to food and water. All animal procedures were reviewed and approved by the Institutional Animal Care and Use Committee (IACUC) of Kaohsiung Veterans General Hospital and were conducted in accordance with institutional guidelines.

### 4.2. Preparation of Stromal Vascular Fractions (SVFs)

Vascular adipose tissue was isolated from bilateral inguinal fat pads of *Lepr*^+/+^, *Lepr^db/db^*, and *Lepr^db/db^* MyD88^−/−^ mice (aged 10–12 weeks). Tissues were minced and digested with collagenase type VIII (Sigma-Aldrich, St. Louis, MO, USA, Cat# C2139) in ice-cold Hank’s Balanced Salt Solution HBSS (2 mg/mL) for 15 min at 37 °C. The digested suspensions were passed through 100 μm cell strainers and centrifuged at 1200 rpm for 10 min. The resulting cell pellets were collected as SVFs. Cell numbers were quantified using a Cellometer (Nexcelom Bioscience, Lawrence, MA, USA). Approximately 2.1 to 2.6 g of adipose tissue was harvested from per *Lepr^db/db^* mouse (average body weight around 42 g).

### 4.3. In Vitro IL-10 Treatment of SVFs

**Figure 11:**
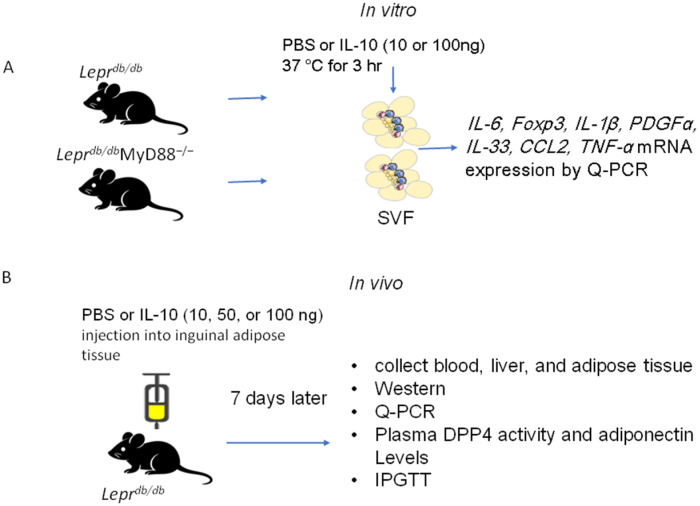
(**A**) For in vitro treatment, SVFs (2 × 10^7^ cells) were harvested from the adipose tissue of *Lepr^db/db^* and *Lepr^db/db^*MyD88^−/−^ mice and treated with recombinant mouse IL-10 (10 or 100 ng) at 37 °C for 3 h. (**B**) For in vivo treatment, IL-10 (10, 50, or 100 ng) or PBS was injected into inguinal adipose tissue. After 7 days, mice were sacrificed, and liver, adipose tissue, and blood samples were collected for further analysis.

For in vitro treatment, SVFs (2 × 10^7^ cells) were suspended in 1 mL PBS and treated with recombinant mouse IL-10 (10, 50, or 100 ng) at 37 °C for 3 h. Cells were then centrifuged at 1700 rpm for 10 min, washed with PBS, and the pellets were collected for subsequent analysis (Figure 11A).

### 4.4. In Vivo IL-10 Injection into Adipose Tissue

*Lepr^+/+^* mice received PBS injections into bilateral inguinal adipose tissue. *Lepr^db/db^* mice (aged 10–12 weeks) were randomly divided into four groups: (I) PBS control; (II) 10 ng IL-10; (III) 50 ng IL-10; and (IV) 100 ng IL-10. IL-10 or PBS was injected directly into inguinal adipose tissue. After 7 days, mice were sacrificed, and liver, adipose tissue, and blood samples were collected for further analysis (Figure 11B).

### 4.5. RNA Isolation and Quantitative Real-Time Polymerase Chain Reaction (Q-PCR)

Total RNA was extracted using Total RNA Miniprep Purification Kits (GeneMark, Atlanta, GA, USA) and reverse-transcribed into cDNA using RT kits (Invitrogen, Carlsbad, CA, USA). For Q-PCR, 2 μL of cDNA (200 ng) was mixed with 12.5 μL of 2× Fast SYBR Green Master Mix (Applied Biosystems, Foster City, CA, USA, Cat# 4385612), 2.5 μL of primers (25 μM each), and 8 μL of sterile water. Amplification was performed using a StepOnePlus™ Real-Time PCR System (Applied Biosystems, Foster City, CA, USA).

### 4.6. Western Immunoblot Analysis

Protein expression of phosphorylated Akt (pAkt; Cell Signaling, #4060), Akt (#4691), JNK (#9252), pJNK (#9251), ERK (#4695), and pERK (#9101) was determined by Western blotting. Tissue samples were homogenized in protein extraction buffer (Sigma-Aldrich, St. Louis, MO, USA) supplemented with a protease inhibitor cocktail (Roche, Basel, Switzerland). Proteins were separated by SDS–PAGE and transferred to nitrocellulose membranes. Membranes were blocked with 5% nonfat milk in TBST buffer (10 mM Tris–HCl, pH 7.5, 150 mM NaCl, 0.1% Tween-20) for 1 h, incubated with primary antibodies for 1 h at room temperature, followed by incubation with secondary antibodies. Protein bands were visualized using enhanced chemiluminescence (ECL; Millipore, Burlington, MA, USA).

### 4.7. Kupffer Cell Purification

Livers were perfused in situ via the portal vein with Ca^2+^- and Mg^2+^-free PBS containing 10 mM EDTA at 37 °C for 5 min, followed by perfusion with HBSS containing 0.1% collagenase IV (Sigma-Aldrich, St. Louis, MO, USA) for 5 min. The liver was excised, dispersed, filtered, and centrifuged at 50× *g* for 1 min at 4 °C. The supernatant was further centrifuged at 300× *g* for 5 min. The resulting cell pellet was layered over a 30/60% Percoll gradient (Pharmacia, Paris, France) and centrifuged at 900× *g* for 15 min to isolate Kupffer cells [23].

### 4.8. Flow Cytometry Analysis

SVFs were suspended in staining buffer (PBS with 0.5% BSA and 2 mM EDTA), incubated with 7-amino-actinomycin D (7-AAD; BioLegend, San Diego, CA, USA), and analyzed using an Attune NxT flow cytometer (ThermoFisher Scientific, Waltham, MA, USA). For intracellular Foxp3 staining, cells were first stained with anti-mouse CD4 (BioLegend, San Diego, CA, USA, #100528), fixed with True-Nuclear™ Fix Buffer for 1 h, and incubated with PE-conjugated anti-mouse Foxp3 antibody (BioLegend, San Diego, CA, USA, #126404) for 1 h in the dark. Data were analyzed using FlowJo v10 software (Tree Star, Ashland, OR, USA).

### 4.9. Insulin Treatment

Mice were injected with either PBS or insulin (1.25 mU/g body weight) and sacrificed 20 min later for liver collection.

### 4.10. Plasma DPP4 Activity

Cardiac blood was collected, and plasma was stored at −20 °C until assayed. Plasma DPP4 activity was measured using a DPP4 Activity Assay Kit (BioVision, Milpitas, CA, USA, # K779-100). Fluorescence of released AMC (7-Amino-4-Methyl Coumarin) was detected at Ex/Em = 360/460 nm.

### 4.11. Serum Alanine Aminotransferase (ALT) and Aspartate Transaminase (AST) Assay

Blood samples were collected from the portal vein, and serum ALT and AST levels were determined using a commercial kit (Transaminase CII-test; Wako Pure Chemical Industries, Osaka, Japan).

### 4.12. Enzyme-Linked Immunosorbent Assay (ELISA)

IL-10 and adiponectin concentrations were determined using mouse ELISA kits (R&D Systems, Minneapolis, MN, USA, #431414; Invitrogen, Carlsbad, CA, USA, #KMP0041). Tissues were homogenized in lysis buffer, and serum samples were obtained by centrifugation. Samples and standards were incubated on antibody-coated plates at 4 °C overnight and detected using HRP-conjugated avidin. IL-10 levels in liver and adipose tissue were normalized to the total protein content.

### 4.13. Intraperitoneal Glucose Tolerance Test (IPGTT)

Following a 15 h fast, mice received intraperitoneal glucose (1 g/kg body weight). Blood glucose levels were measured at 0, 15, 30, 45, 60, 75, 90, and 120 min post-injection using a glucose meter (Accu-Chek Performa; Roche, Basel, Switzerland).

### 4.14. Statistical Analysis

Data are presented as mean ± standard error of the mean (SEM). Statistical analyses were conducted with GraphPad Prism 10.0 software. Statistical significance was determined using unpaired Student’s *t*-tests for two-group comparisons or one-way analysis of variance (ANOVA) with Tukey’s post hoc test for multiple comparisons. A *p* value < 0.05 was considered statistically significant.

## 5. Conclusions

**Figure 12:**
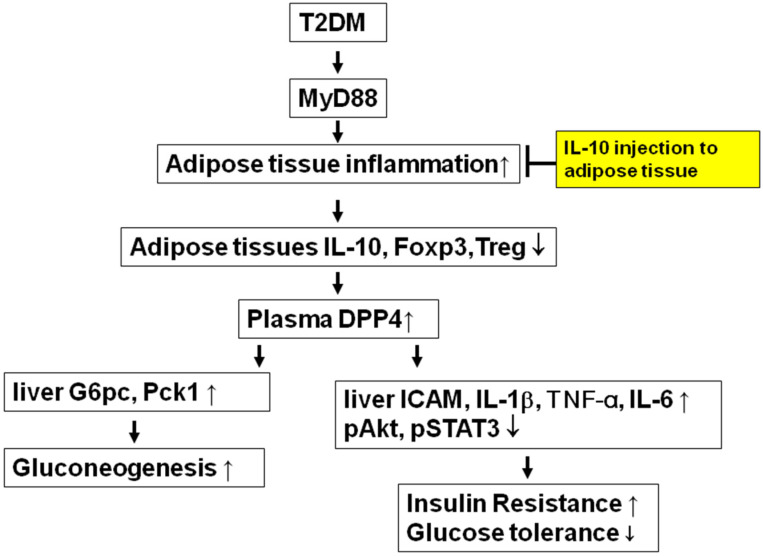
MyD88 signaling plays a pivotal role in T2DM-induced adipose inflammation, hepatic inflammation, and gluconeogenesis by suppressing adipose-derived IL-10 and Foxp3^+^ regulatory T cells. Injection of IL-10 in adipose tissue reduced adipose inflammation, attenuated hepatic inflammation and gluconeogenesis, improved insulin signaling, and alleviated glucose intolerance in diabetic mice.

In conclusion, our study demonstrates that MyD88 signaling plays a pivotal role in promoting adipose inflammation, hepatic inflammation, and gluconeogenesis in T2DM by suppressing adipose-derived IL-10 and Foxp3^+^ regulatory T cells. Genetic deletion of MyD88 or pharmacological elevation of IL-10 in adipose tissue significantly reduced adipose IL-6 and CCL2 expression, attenuated hepatic inflammation and gluconeogenesis, improved insulin signaling, and alleviated glucose intolerance in diabetic mice (Figure 12). These findings identify the adipose MyD88/IL-10/Treg axis as a critical mediator of adipose–liver crosstalk in metabolic disease and suggest that targeting this pathway may represent a novel therapeutic strategy for the treatment of metabolic syndrome and T2DM.

## Data Availability

The original contributions presented in this study are included in the article/Supplementary Material. Further inquiries can be directed to the corresponding author.

## Supplementary Materials

The following supporting information can be downloaded at: https://www.mdpi.com/article/10.3390/ijms27062883/s1.

## Abbreviation

DM	Diabetes mellitus
DKA	Diabetic ketoacidosis
SVFs	Stromal vascular fractions
PCK1	Cytosolic form of Phosphoenolpyruvate carboxykinase
G6PC	Glucose 6-phosphatase catalytic subunits
TNF-α	Tumor Necrosis Factor-α
IL-1β	Interleukin-1β
IL-33	Interleukin-33
CCL2	Monocyte chemoattractant protein-1
iNOS	Inducible nitric oxide synthase
DPP4	Dipeptidyl peptidase-4
ALT	Alanine aminotransferase
AST	Aspartate transaminase
IL-10	Interleukin-10
Foxp3	Forkhead box protein P3
MSC	Mesenchymal stem cells
FGF21	Fibroblast growth factor 21
ATM	Adipose tissue macrophage
NATM	Non-adipose tissue macrophage
PDGFα	Platelet-derived growth factor
STAT3	signal transducer and activator of transcription 3
mTOR	mammalian target of tapamycin
WAT	white adipose tissue
MyD88	Myeloid differentiation factor 88
