# Supplementary material for: MyD88 Inhibition Ameliorates Diabetes-Induced Hepatic Inflammation and Gluconeogenesis Through Adipose IL-10 Induction
Source: Int J Mol Sci. 2026 Mar 23;27(6):2883. doi: 10.3390/ijms27062883 (PMC13026309; doi:10.3390/ijms27062883)
Supplement: Supplementary file 1 [file ijms-27-02883-s001.zip › ijms-4107587-supplementary.pdf]

MyD88 inhibition ameliorates diabetes-induced hepatic inflammation and gluconeogenesis through adipose IL-10 induction

Yi-Cheng Li[1], Hsiao-Chi Lai[2,3], Pei-Hsuan Chen[2,4], Chia-Hua Tang[2], Lee-Wei Chen[2,4,5*]

Supplemental Figures

Supplemental Figure S1:Adipose tissue was harvested from *Lepr*$^{+/+}$,*Lepr*$^{db/db}$, and *Lepr*$^{db/db}$MyD88−/− mice to investigate the effect of MyD88 signaling depletion in the protein expression of FOXp3 in adipose tissue. Uncropped Western blot images of FOXp3 of SVFs from adipose tissue. Representative images and statistical analysis are presented in Figure 1A.

Foxp3->

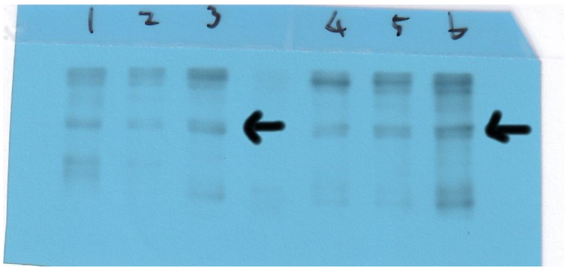

Foxp3->

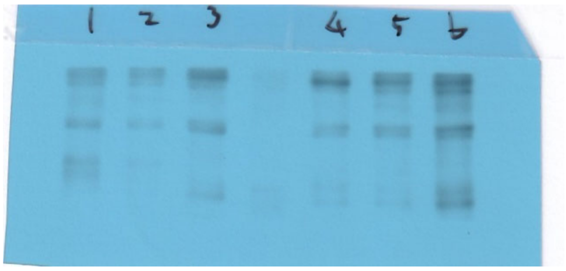

β-actin->

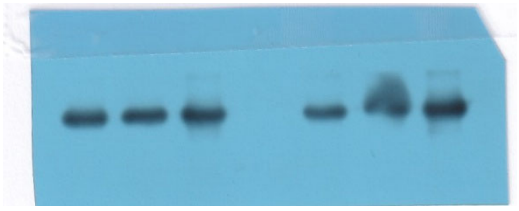

Lepr$^{+/+}$ Lepr$^{db/db}$ Lepr$^{db/db}$MyD88$^{-/-}$ Lepr$^{+/+}$ Lepr$^{db/db}$ Lepr$^{db/db}$MyD88$^{-/-}$

Supplemental Figure S2:PBS or 10 ng of IL-10 were injected into adipose tissue of T2DM (*Lepr*$^{db/db}$) mice to investigate the involvement of IL-10 in the decreased inflammatory cytokine expression in adipose tissue. Uncropped Western blot images of pJNK, p-NF-kB, JNK, and b-actin of SVFs of adipose tissue. Representative images and statistical analysis are presented in Figure 5B.

pJNK->

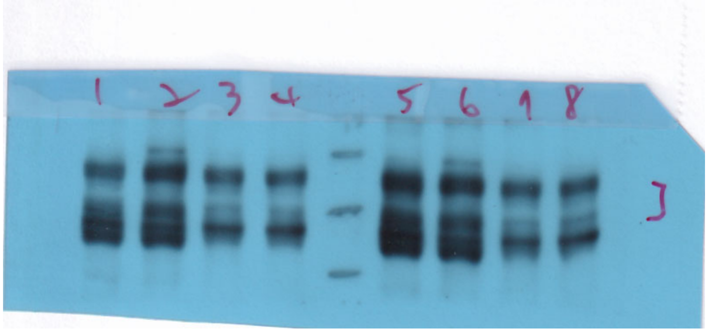

pJNK->

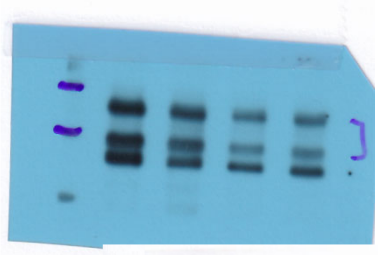

JNK->

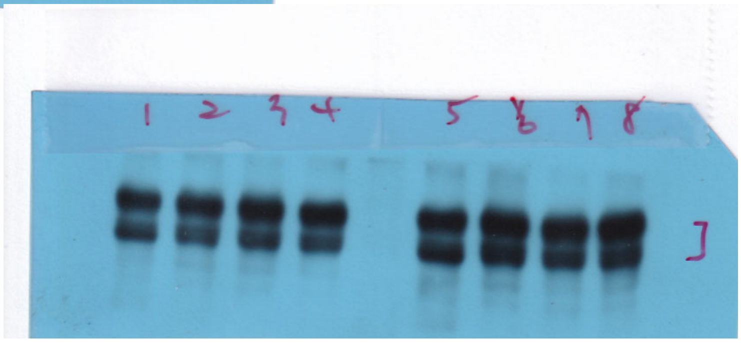

JNK->

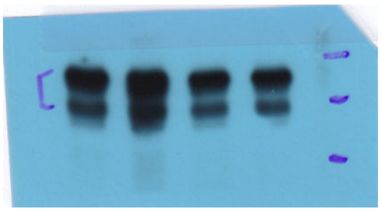

p-NF-kB→

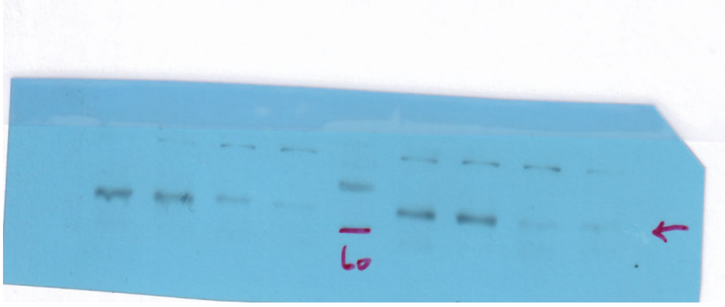

NF-kB→

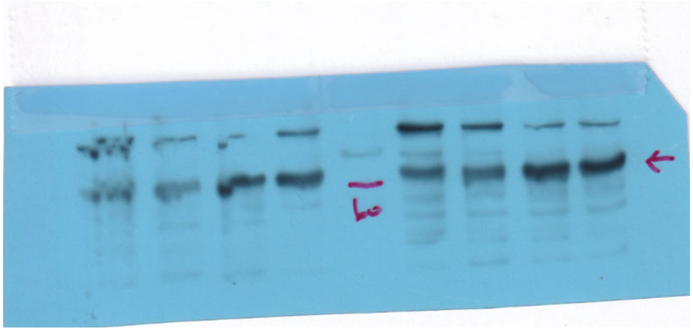

β-actin->

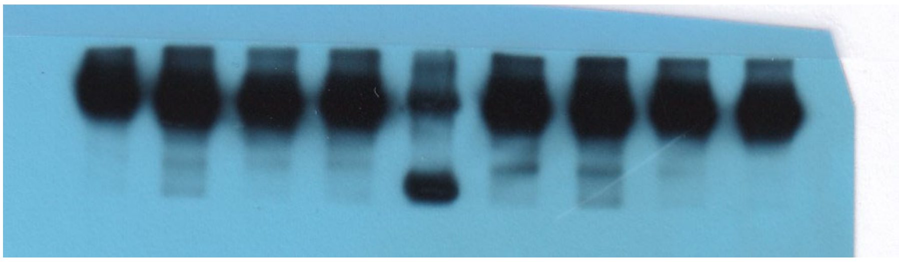

Lepr$^{+/+}$	PBS	10 ng	100 ng	Lepr$^{+/+}$	PBS	10 ng	100 ng
		IL-10				IL-10	
	Lepr$^{db/db}$				Lepr$^{db/db}$		

Supplemental Figure S3:PBS or 10, 50, and 100 ng of IL-10 were injected into adipose tissue of T2DM (*Lepr*$^{db/db}$) mice, and the liver was harvested 7 days after injection to further examine whether IL-10 injection decreased inflammatory cytokine protein exsression of the liver.Uncropped Western blot images of pAkt, Akt, pERK, and pSTAT3 of liver. Representative images and statistical analysis are presented in Figure 8B.

Akt->

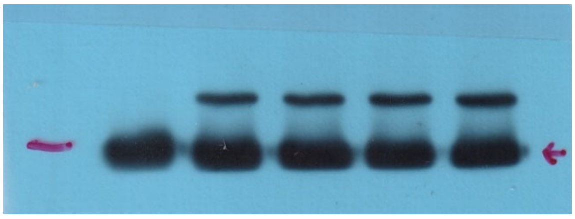

Akt->

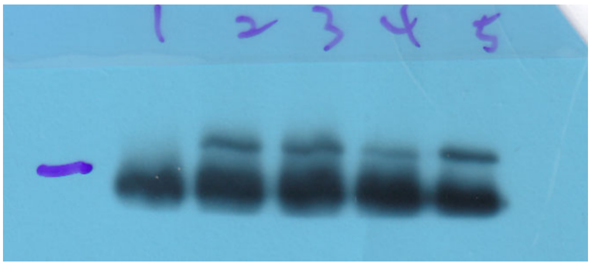

Akt->

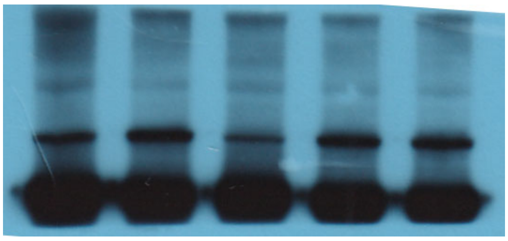

pAkt->

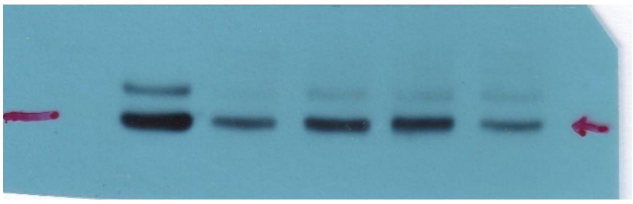

pAkt->

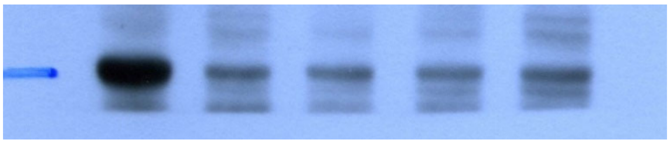

pAkt->

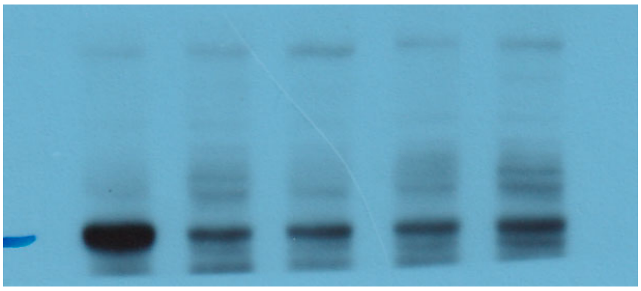

PBS PBS 10 ng 50 ng 100 ng

IL-10

Lepr$^{+/+}$ *Lepr*$^{db/db}$

ERK->

p-ERK->

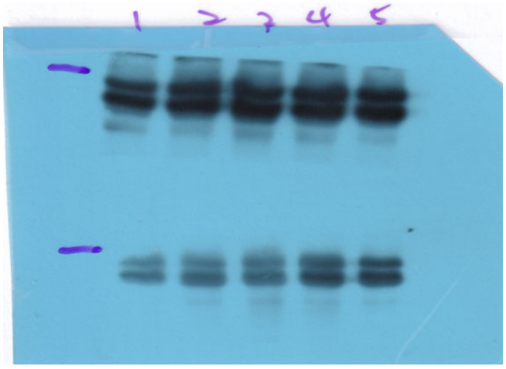

ERK->

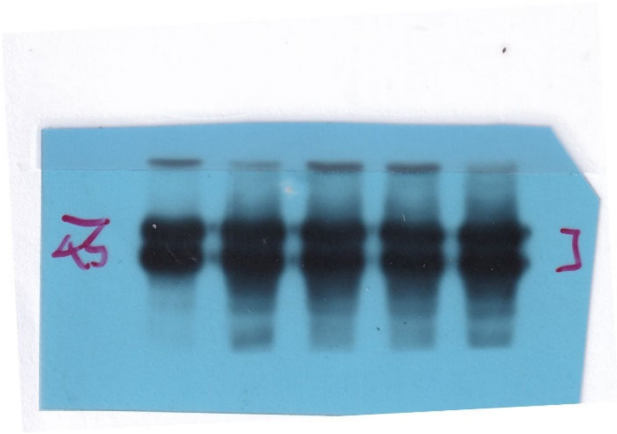

p-ERK->

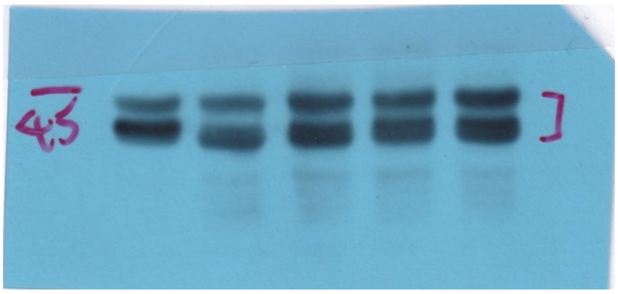

pSTAT3

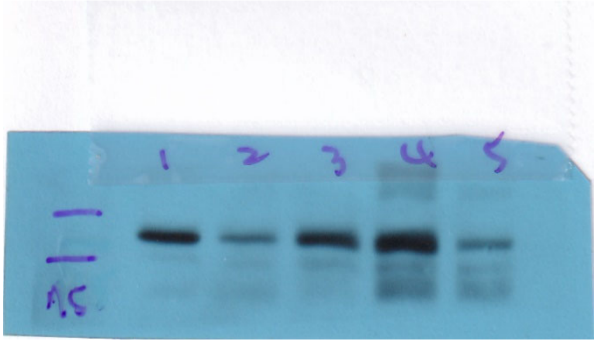

pSTAT3

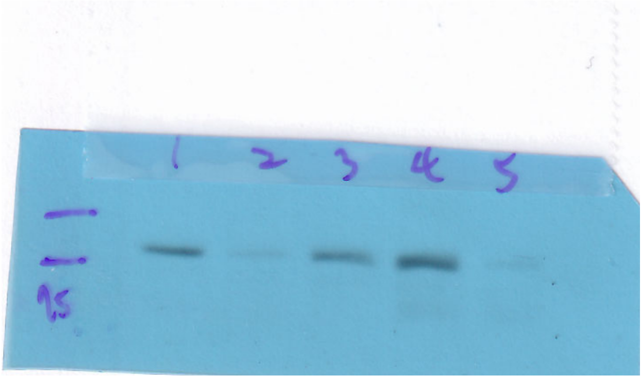

pSTAT3

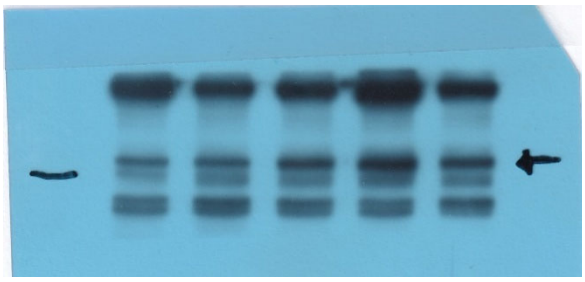

STAT3->

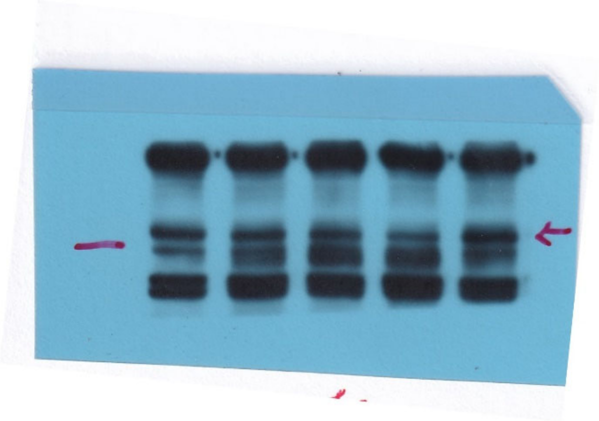

PBS PBS 10 ng 50 ng 100 ng

IL-10

Lepr$^{+/+}$ **_Lepr_**$^{db/db}$

β-actin->

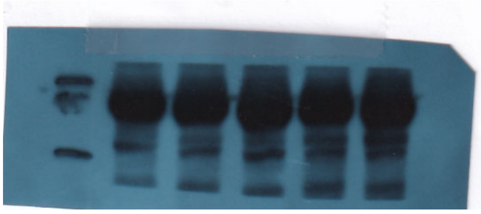

β-actin->

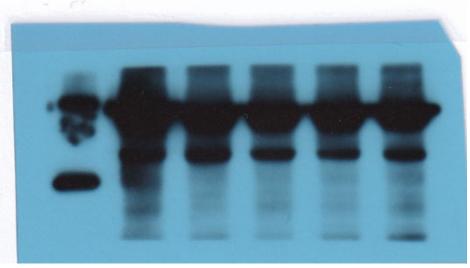

PBS PBS 10 ng 50 ng 100 ng

IL-10

Lepr$^{+/+}$ **_Lepr_**$^{db/db}$

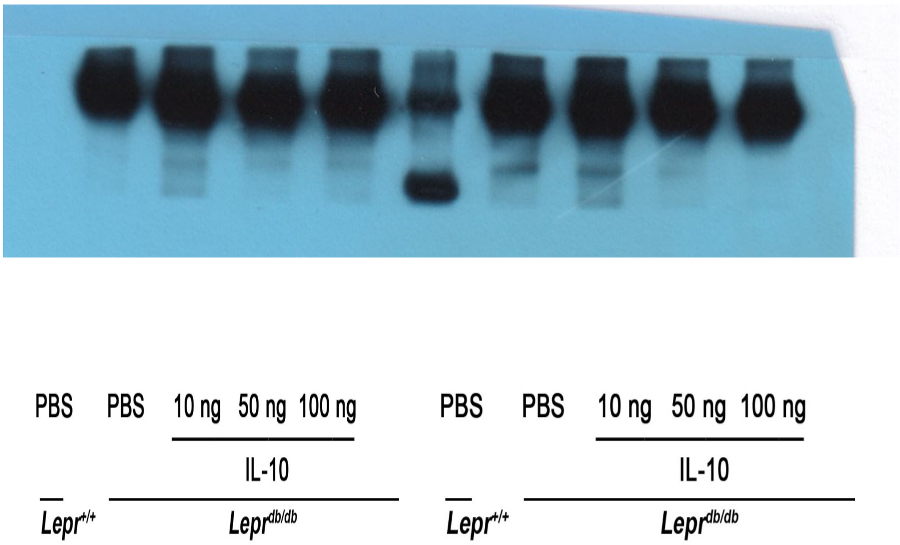
PBS
PBS
10 ng
50 ng
100 ng
IL-10
Lepr+/+
Leprdb/db
PBS
PBS
10 ng
50 ng
100 ng
IL-10
Lepr+/+
Leprdb/db

Supplemental Figure S4:$Lepr^{db/db}$ mice were injected with PBS or 10 ng or 50 ng of IL-10 followed by insulin administration, and the protein expression of Akt phoshorylation in the liver of $Lepr^{db/db}$ mice was then measured to investigate whetherIL-10injection attenuatesinsulin resistance in diabetic mice. Uncropped Western blot images of pAkt and Akt of liver. Representative images and statistical analysis are presented in Figure 10B.

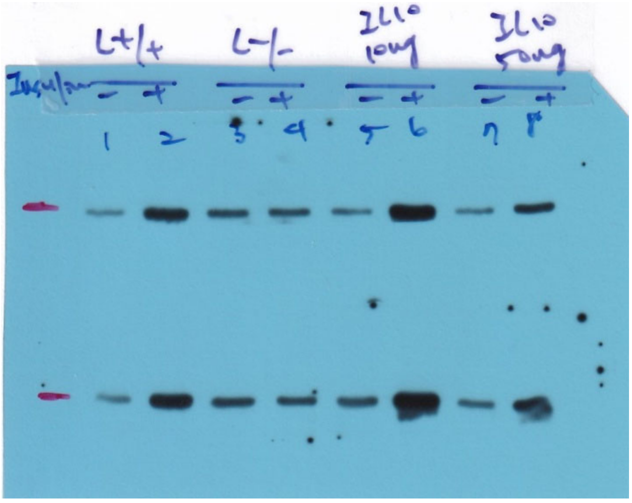

pAkt->

pAkt->

$Lepr^{+/+}$ $Lepr^{db/db}$ $Lepr^{db/db}$ $Lepr^{db/db}$

IL-10 10 ng IL-10 50 ng

Insulin - + - + - + - +

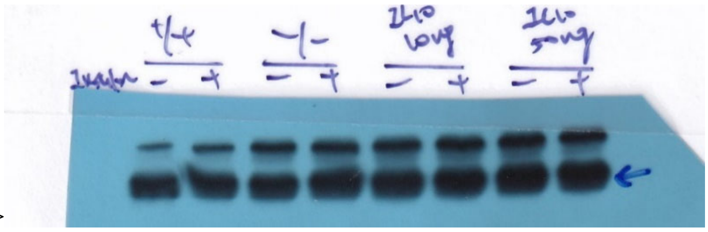

Akt->
